# Molecular characterization and PCR-based replicon typing of multidrug resistant *Shigella sonnei* isolates from an outbreak in Thimphu, Bhutan

**DOI:** 10.1186/1756-0500-7-95

**Published:** 2014-02-20

**Authors:** Sirigade Ruekit, Sonam Wangchuk, Tshering Dorji, Kinzang Pem Tshering, Piyarat Pootong, Panida Nobthai, Oralak Serichantalergs, Kamonporn Poramathikul, Ladaporn Bodhidatta, Carl Jeffries Mason

**Affiliations:** 1 Department of Enteric Diseases, Armed Forces Research Institute of Medical Sciences (AFRIMS), Bangkok, Thailand; 2 Public Health Laboratory, Department of Public Health, Ministry of Health, Royal Government of Bhutan, Thimphu, Bhutan; 3 Department of Pediatrics, Jigme Dorji Wangchuk National Referral Hospital, Kawa Jangsa, Thimphu, Bhutan

**Keywords:** *Shigella sonnei*, Multidrug resistance, Bhutan

## Abstract

**Background:**

*Shigella* species are an important cause of diarrhea in developing countries. These bacteria normally acquire their antibiotic resistance via several different mobile genetic elements including plasmids, transposons, and integrons involving gene cassettes. During a diarrhea surveillance study in Thimphu, Bhutan in June and July, 2011, *Shigella sonnei* were isolated more frequently than expected. This study describes the antibiotic resistance of these *S. sonnei* isolates.

**Methods:**

A total of 29 *S. sonnei* isolates from Thimphu, Bhutan was characterized for antimicrobial susceptibility by disc diffusion assay and minimum inhibitory concentration (MIC) assay. All isolates were tested by pulsed-field gel electrophoresis (PFGE) with restriction enzyme *Xba*I and were tested for plasmid. The plasmid patterns and the PFGE patterns were analyzed by Bionumerics software. DNA sequencing was performed on amplified products for *gyraseA* gene and class 1 and class 2 integrons. *S. sonnei* isolates were classified for incompatibility of plasmids by PCR-based replicon typing (PBRT).

**Results:**

These *S. sonnei* were resistant to multiple drugs like ciprofloxacin, nalidixic acid, trimethoprim-sulfamethoxazole, streptomycin, and tetracycline but susceptible to azithromycin. All isolates had class 2 integrons *dfrA1, sat1* and *aadA1* genes. Two point mutations in Gyrase A subunit at position Ser83Leu and Asp87Gly were detected in these quinolone resistant isolates. The plasmid and PFGE patterns of *S. sonnei* isolates suggested a clonal relationship of the isolates. All isolates carried common ColE plasmid. ColE plasmid co-resided with B/O plasmid (nine isolates) or I1 plasmid (one isolate).

**Conclusions:**

The characteristics of 29 *S. sonnei* isolates from Thimphu, Bhutan in June and July, 2011 are identical in PFGE, plasmid and resistance pattern. This study suggests that these recent *S. sonnei* isolates are clonally related and multidrug-resistant.

## Background

*Shigella* species are an important cause of diarrhea in developing countries. In 2013, the World Health Organization (WHO) reported the diarrheal disease is a leading cause of child mortality and morbidity in the world, and mostly results from contaminated food and water sources. Worldwide, 780 million individuals lack access to improved drinking-water and 2.5 billion lack improved sanitation. Diarrhea due to infection is widespread throughout developing countries. Globally, there are nearly 1.7 billion cases of diarrheal disease every year. Each year diarrhea kills around 760, 000 children under five years of age [1]. Bhutan is a developing country in the South Asia region with decent health care access throughout the country. The current population of the country is around 706,800 with about 80% of the population residing in rural areas. Children under 5 years of age comprise about 10% of the total population with an annual birth cohort of 14,000 [2]. For Bhutan, diarrheal disease is an important public health problem. Treatment for diarrheal disease in the country is empirical rather than evidence based due to the lack of diarrhea etiology data. In the Annual Health Bulletin, Bhutan reported an average of 60,000-70,000 cases of diarrhea and 25,000-30,000 cases of dysentery per year from 2005–2009. The overall incidence of diarrheal diseases in Bhutan during 2010 was 126.6 per 1,000 people. The percentage of diarrhea and dysentery that occurred in children less than 5 years of age for 2009 were 34% and 30%, respectively [3]. Shigellosis has been a notifiable disease in Bhutan since May 2010 with only one case reported [4]. In 2011, a reported diarrhea outbreak probably caused by *Shigella flexneri* occurred during March 2011 in a remote village in Mongar District, Bhutan [5]. During a diarrhea surveillance study in Thimphu, Bhutan from March 2011 to February 2013, *Shigella sonnei* was isolated from children’s stool samples more frequently than expected in June and July 2011.

The rapid emergence of multidrug-resistant (MDR) strains is largely due to their ability to acquire and disseminate exogenous genes associated with mobile genetic elements such as, transposons, integrons, plasmids, and genomic islands [6]. Some transposons code for single resistance [e.g. TEM b-lactamase (Tn*3*), kanamycin (Tn*5*) and tetracycline resistance (Tn*10*)], but plasmids and transposons coding multiple drug resistance often possess another genetic element, the integron [7]. Integrons are genetic elements that contain the components of a site-specific recombination system that recognizes and captures mobile gene cassettes. Based on the characteristics of their integrase genes, four classes of integrons (classes 1, 2, 3, and 4) have been identified to date [8]. Class 1 and 2 integrons have been frequently reported in studies of human *Shigella*[9]. Antibiotic-resistance plasmids frequently contain genes conferring resistance to several different antibiotics. The ability to recognize and categorize plasmids in homogeneous groups on the basis of their phylogenetic relatedness can be helpful to analyze their distribution in nature, the relationship with the host cell and to discover their evolutionary origin [10].

To further study the multidrug resistance in Thimphu, Bhutan, we characterized *S. sonnei* isolates from children, the genetic basis of antibiotic resistance including class 1 and class 2 integrons, DNA gyrase A mutations and plasmid replicon typing.

## Methods

### Clinical specimens

A diarrhea etiology study was conducted at Jigme Dorji Wangchuk National Referral Hospital (JDWNRH) in Thimphu, Bhutan from March 2011 to February 2013. Children 3 months to 5 years of age presenting for clinical care at JDWNRH with a chief complaint of acute diarrhea (3 or more loose stools in the previous 24 hours starting no more than 72 hours before presenting were eligible to provide a stool sample as cases. Children 3 months to 5 years presenting for clinical care at JDWNRH for other causes, who not had a history of diarrhea in the past 2 weeks and had not received antibiotics in the past 3 days were eligible to provide stool samples as controls. The study was approved by the Research Ethics Board of Health (REBH), Bhutan and the Institutional Review Board (IRB), Walter Reed Army Institute of Research (WRAIR). Stool samples were collected from children with verbal informed consent from their parent or guardian.

### Specimen processing and identification

Stool samples collected in Cary-Blair transport medium were cultured on MacConkey (MAC) and Hektoen Enteric (HE) agar (Becton-Dickinson and Company, Sparks, MD, USA) and subsequently incubated at 37°C overnight. Plates are examined for the presence of colonies resemble to *Shigella* and identified by standard biochemical tests and further serotyped by agglutination test (Denka Seiken, Japan) at the Armed Forces Research Institute of Medical Sciences (AFRIMS) in Bangkok, Thailand [11]. Isolates were stored in Luria-Bertani (LB) broth containing 15% glycerol at -70°C until use.

### Antimicrobial susceptibility methods

The *S. sonnei* isolates were tested for susceptibility to antimicrobial agents by the standard disk diffusion method according to Clinical and Laboratory Standards Institute (CLSI) guideline [12]. Antimicrobial disks included azithromycin (AZM 15 μg), nalidixic acid (NA 30 μg), ciprofloxacin (CIP 5 μg), ampicillin (AM 10 μg), trimethoprim-sulfamethoxazole (SXT 1.25/23.75 μg), ceftriaxone (CRO 30 μg), streptomycin (S 10 μg), and tetracycline (TE 30 μg) (Sensi-Disc; Becton Dickinson, NJ, USA). Minimum inhibitory concentrations (MIC) for AZM, NA, and CIP were also determined by E-test (AB Biodisk, Solna, Sweden). The standard disk diffusion method and MIC were interpreted according to CLSI guideline [12]. Organisms used for quality control of antimicrobial susceptibility testing included *Escherichia coli* ATCC 25922, *Staphylococcus aureus* ATCC 25923, and *Staphylococcus aureus* ATCC 29213. In the absence of CLSI definitive standards for azithromycin (AZM) breakpoints of the family Enterobacteriaceae, interpretive standards as published by the disk manufacturer for *Staphylococcus* spp. was used (BBL package insert; Becton-Dickinson and Company, Sparks, MD, USA). Multidrug-resistant (MDR) *S. sonnei* strains were defined as strains that were resistant to two or more antimicrobial agents.

### Pulsed-field gel electrophoresis (PFGE)

Pulsed-field gel electrophoresis (PFGE) was performed according to the One-Day (24–28 h) Standardized Laboratory Protocol for Molecular Subtyping by PFGE [13] with minor modifications. The cell density of each isolate was adjusted by spectrophotometer (Bio-Rad, Hercules, CA, USA). Plug slices of samples and *Salmonella* Braenderup H9812 universal size standard were prepared for *Xba*I digestion and loaded on 1% SeaKem Gold agarose (Lonza, Rockland, ME, USA) with 0.5x TBE pH 8.3 (890 mM Tris, 890 mM Borate, 20 mM EDTA). The gel was electrophoresed by CHEF Mapper system (Bio-Rad, Hercules, CA, USA). The electrophoresis conditions for *Xba*I digestion were auto algorithm mode, 30–600 kb for molecular weight range, and 19 h for run time. The gel images were captured by a gel documentation system (Syngene, Cambridge, UK). Combined dendrogram of PFGE and antimicrobial susceptibility were generated by BioNumerics Software version 6.0 (Applied Maths, Kortrijk, Belgium), UPGMA (Unweighted Pair Group Method with Arithmetic Mean) type and Dice coefficient with 1.0% optimization and tolerance.

### Plasmid analysis

The *S. sonnei* isolates, *E. coli* V.517 and *E. coli* 39R were grown in 5 ml LB broth at 200 rpm, 37°C overnight. Plasmid DNAs were extracted by alkaline lysis method [14] and separated by electrophoresis on a 0.8% agarose gel with 0.5x TBE using plasmids from *E. coli* V.517 and *E. coli* 39R as reference size markers. A combined dendrogram of plasmid and antimicrobial susceptibility were generated by BioNumerics Software version 6.0 (Applied Maths, Kortrijk, Belgium), UPGMA type and Dice coefficient with 1.5% optimization and tolerance.

### Multiplex PCR for plasmid replicon typing

The *S. sonnei* isolates were examined by PCR using three multiplex primer panels for the presence of 18 replicon plasmids and three singleplex primer pairs for an additional 3 replicon plasmids, ColE, IncU and IncR plasmids (Table 1). *S. sonnei* isolates and reference plasmids for controls were grown on TSA plates at 37°C overnight and were extracted for genomic DNA using DNeasy Blood and Tissue Kit (QIAGEN, Valencia, CA, USA). The reference plasmid controls were kindly provided by Dr. Alessandra Carottoli. PCR reactions were performed as previously described [15,16]. Positive controls as well as a negative control without DNA were run with each multiplex primer panel.

**Table 1:** PCR primer sequence

**Primer**	**Sequence (5’-3’)**	**Location**	**Annealing temperature (°C)**	**Product size (bp)**	**Reference**
IntF	GGCATCCAAGCAGCAAGC	5’-CS of class 1 integrons	58	varied	[17]
IntB	AAGCAGACTTGACCTGAT	3’-CS of class 1 integrons			
IntI2L	GTAGCAAACGAGTGACGAAATG	intI2	58	789	[17]
IntI2R	CACGGATATGCGACAAAAAGGT				
IntI2CaF	GATAAAAACAGCCTGACCTCTTC	intI2	58	2433	[17]
IntI2CaR	CCCACTTGACATCTCATCAATAC	3’ region of class 2 integrons			
ST.GYRA1	TGTCCGAGATGGCCTGAAGC	DNA gyrase A	55	470	[18]
ST.GYRA12	CGTTGATGACTTCCGTCAG				
*mphA*F	AACTGTACGCACTTGC	*macrolide* 2’-phosphotransferase	50	837	[19]
*mphA*R	GGTACTCTTCGTTACC				
**Multiplex 1**					
B/O_F	GCGGTCCGGAAAGCCAGAAAAC	RNAI	60	159	[15]
B/O_R	TCTGCGTTCCGCCAAGTTCGA				
FIC_F	GTGAACTGGCAGATGAGGAAGG	repA2	60	262	[15]
FIC_R	TTCTCCTCGTCGCCAAACTAGAT				
A/C_F	GAGAACCAAAGACAAAGACCTGGA	repA	60	465	[15]
A/C_R	ACGACAAACCTGAATTGCCTCCTT				
P_F	CTATGGCCCTGCAAACGCGCCAGAAA	iterons	60	534	[15]
P_R	TCACGCGCCAGGGCGCAGCC				
T_F	TTGGCCTGTTTGTGCCTAAACCAT	repA	60	750	[15]
T_R	CGTTGATTACACTTAGCTTTGGAC				
**Multiplex 2**					
K/B_F	GCGGTCCGGAAAGCCAGAAAAC	RNAI	60	160	[15]
K/B_R	TCTTTCACGAGCCCGCCAAA				
W_F	CCTAAGAACAACAAAGCCCCCG	repA	60	242	[15]
W_R	GGTGCGCGGCATAGAACCGT				
FIIA_F	CTGTCGTAAGCTGATGGC	repA	60	270	[15]
FIIA_R	CTCTGCCACAAACTTCAGC				
FIA_F	CCATGCTGGTTCTAGAGAAGGTG	iterons	60	462	[15]
FIA_R	GTATATCCTTACTGGCTTCCGCAG				
FIB_F	GGAGTTCTGACACACGATTTTCTG	repA	60	702	[15]
FIB_R	CTCCCGTCGCTTCAGGGCATT				
Y_F	AATTCAAACAACACTGTGCAGCCTG	repA	60	765	[15]
Y_R	GCGAGAATGGACGATTACAAAACTTT				
**Multiplex 3**					
I1_F	CGAAAGCCGGACGGCAGAA	RNAI	60	139	[15]
I1_R	TCGTCGTTCCGCCAAGTTCGT				
Frep_F	TGATCGTTTAAGGAATTTTG	RNAI/repA	60	270	[15]
Frep_R	GAAGATCAGTCACACCATCC				
X_F	AACCTTAGAGGCTATTTAAGTTGCTGAT	ori γ	60	376	[15]
X_R	TGAGAGTCAATTTTTATCTCATGTTTTAGC				
HI1_F	GGAGCGATGGATTACTTCAGTAC	parA-parB	60	471	[15]
HI1_R	TGCCGTTTCACCTCGTGAGTA				
N_F	GTCTAACGAGCTTACCGAAG	repA	60	559	[15]
N_R	GTTTCAACTCTGCCAAGTTC				
HI2_F	TTTCTCCTGAGTCACCTGTTAACAC	iterons	60	644	[15]
HI2_R	GGCTCACTACCGTTGTCATCCT				
L/M_F	GGATGAAAACTATCAGCATCTGAAG	repA, B, C	60	785	[15]
L/M_R	CTGCAGGGGCGATTCTTTAGG				
oricolE_F	GTTCGTGCATACAGTCCA	ColE plasmids	60	187	[16]
oricolE_R	GGCGAAACCCGACAGGAC				
IncR_F	TCGCTTCATTCCTGCTTCAGC	IncR plasmids	60	251	[16]
IncR_R	GTGTGCTGTGGTTATGCCTCA				
IncU_F	TCACGACACAAGCGCAAGGG	IncU plasmids	60	843	[16]
IncU_R	TCATGGTACATCTGGGCGC				

### Amplification of Integrons, DNA gyrase A gene and azithromycin resistance gene (*mphA*)

The *S. sonnei* isolates were grown on TSA plates at 37°C overnight and purified for genomic DNA extracted using DNeasy Blood and Tissue Kit (QIAGEN, Valencia, CA, USA). DNA templates were kept at -20°C before use. Extracted DNA was amplified for class 1 and class 2 integrons, DNA gyrase A gene and azithromycin resistance gene (*mphA*) by PCR (Table 1), as previously described [17-19].

### Restriction fragment length polymorphism (RFLP)

RFLP was performed on amplified DNA of class 2 integrons of the *S. sonnei* isolates by using Int2CaF and Int2CaR primers (Table 1) and PCR conditions as previously described [17]. Amplified product was purified by Wizard® SV Gel and PCR Clean-Up System (Promega, Madison, WI, USA) and digested with *Xba*I (Roche, Indianapolis, IN, USA) at 37°C for 2 hours. The restriction fragments were detected on a 1.5% agarose gel by electrophoresis with 0.5X TBE buffer. Two-log DNA ladder (BioLabs, Ipswich, MA, USA) was used as a standard DNA marker.

### Sequencing and bioinformatics analysis

The amplified PCR products of *S. sonnei* isolates, class 1 integrons, class 2 integrons and the gyrase A gene were purified by Wizard® SV Gel and PCR Clean-Up System and were sequenced (Macrogen, Korea). The nucleotide sequences were analyzed with BLAST software (http://www.ncbi.nlm.nih.gov/BLAST).

## Results

### *Shigella sonnei* isolates

**Figure 1:**
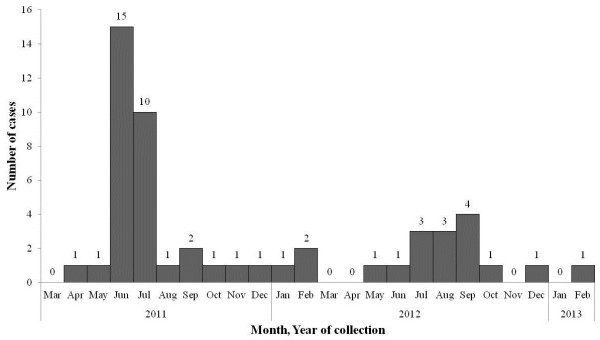
**Epidemic curve of *S. sonnei *** **isolates in Thimphu, Bhutan from March 2011 to February 2013.**

During the surveillance study from March 2011 to February 2013 at JDWNRH in Thimphu, Bhutan, a total of 1,133 children from 3 months to 5 years of age were studied. This included 571 children with diarrhea and 562 non-diarrhea controls. The percentage of *Shigella* spp*.* detected were 70.9% (56/79) of *S. sonnei*, 19.0% (15/79) of *S. flexneri*, 8.9% (7/79) of *S. boydii* and 1.2% (1/79) of *S. dysenteriae*. An epidemic curve of the number of cases per month of *S. sonnei* from March 2011 to February 2013 was created, a total of 56 *S. sonnei* isolates were 51 isolates from cases and 5 isolates from controls (Figure 1). During the apparent outbreak of *S. sonnei* in Thimphu, Bhutan in June and July 2011, a total of 25 *S. sonnei* were isolated from cases and 4 *S. sonnei* were isolated from asymptomatic controls. The other 27 *S. sonnei* isolates were sporadically found from March 2011 to February 2013. All 29 *S. sonnei* isolates during the outbreak period were characterized in this study. Four *S. sonnei* from Nepal in 2007–2008 and five *S. sonnei* from Thailand in 2008 isolated previously at AFRIMS were included in this study for comparison.

### Multidrug-resistant *Shigella sonnei* in Bhutan

In this study, the 29 *S. sonnei* isolates from Thimphu, Bhutan were resistant to NA, CIP, SXT, S and TE by disk diffusion assay (Table 2). These 29 isolates had an MIC of NA (>256 μg/ml) and CIP (3.0-4.0 μg/ml) by E-test confirming the resistance described by disk diffusion assay. All 29 isolates were susceptible to AZM by disk diffusion assay. The MIC range of AZM by E-test was 3.0-4.0 μg/ml corresponding to intermediate susceptibility to AZM (Table 2).

**Table 2:** **Characteristics of *S. sonnei *** **isolates from Bhutan, Nepal and Thailand**

**Study site**	**Number of isolates**	**Resistance pattern**	**MIC (μg/ml)**			**Integrons**	**DNA Gyrase A mutation**		**Replicon plasmid**
			**AZM**	**NA**	**CIP**		**Ser83**	**Asp87**	
Bhutan	19	S, SXT, TE, NA, CIP	3.0-4.0	>256	3.0-4.0	Class 2	Ser83Leu	Asp87Gly	ColE
Bhutan	9	S, SXT, TE, NA, CIP	3.0-4.0	>256	3.0-4.0	Class 2	Ser83Leu	Asp87Gly	B/O, ColE
Bhutan	1	S, SXT, TE, NA, CIP	4.0	>256	3.0	Class 2	Ser83Leu	Asp87Gly	I1, ColE
Nepal	1	S, SXT, TE, NA, CIP	4.0	>256	4.0	Class 2	Ser83Leu	Asp87Gly	ColE
Nepal	2	S, SXT, TE, NA	3.0-4.0	>256	0.190-0.125	Class 2	Ser83Leu	Neg	ColE
Nepal	1	S	3.0	3.0	0.008	Neg	Neg	Neg	ColE
Thailand	1	S, SXT, TE, NA, AM	3.0	>256	0.190	Neg	Ser83Leu	Neg	B/O, ColE
Thailand	1	S, SXT, TE, AM	3.0	3.0	0.004	Neg	Neg	Neg	I1, ColE
Thailand	2	S, SXT, TE, NA, AM	3.0-4.0	128	0.004	Class 2	Neg	Asp87Tyr	I1, ColE
Thailand	1	S, SXT, TE, NA,	4.0	>256	0.004	Class 2	Ser83Leu	Neg	ColE

AZM, azithromycin; NA, nalidixic acid; CIP, ciprofloxacin; AM, ampicillin; SXT, trimethoprim-sulfamethoxazole; CRO, ceftriaxone; S, streptomycin; TE, tetracycline; Ser, Serine; Leu, Leucine; Asp, Aspartic acid; Gly, Glycine; Tyr, Tyrosine; Neg, negative result.

### PFGE patterns and plasmid patterns analysis

**Figure 2:**
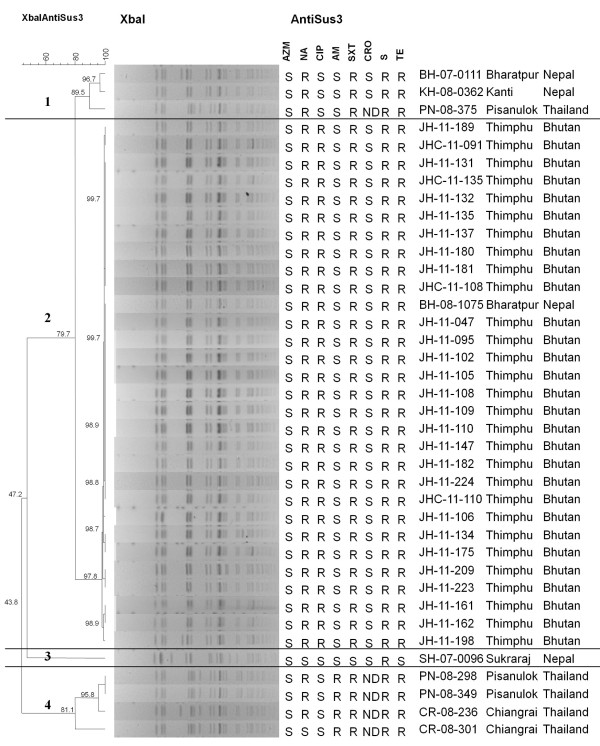
**Dendrogram obtained by cluster analysis of the PFGE patterns of *S. sonnei *from Bhutan, Nepal and Thailand combined with antibiotic susceptibility.** AZM, azithromycin; NA, nalidixic acid; CIP, ciprofloxacin; AM, ampicillin; SXT, trimethoprim-sulfamethoxazole; CRO, ceftriaxone; S, Streptomycin; TE, tetracycline; S, susceptible; R, resistant; ND, not determined.

At 80% similarity, the PFGE patterns of all 38 isolates clustered into 4 groups (Figure 2). Groups 1, 3 and 4 included *S. sonnei* from Nepal and Thailand. Group 2 consisted of the 29 *S. sonnei* isolates from Bhutan and 1 isolate from Nepal. *S. sonnei* isolates from Bhutan were closely related within the subgroup with 97-100% similarity. All 30 isolates in group 2 had a pattern of resistance to NA, CIP, SXT, S and TE (Figure 2).

*S. sonnei* from Bhutan contained various plasmids with sizes ranging from 0.35 Kb to 98 Kb. Nine isolates from Bhutan contained larger plasmids sized from 32 Kb to 98 Kb similar to 1 isolate from Nepal and 4 isolates from Thailand. Most isolates from all three sites contained variable plasmids sized from 0.35 Kb to 25 Kb. At 62% similarity, dendrogram of the plasmid DNA patterns of *S. sonnei* was classified into 4 groups. Group 2 consisted of the 29 *S. sonnei* isolates from Bhutan and 1 isolate from Nepal. Groups 1, 3 and 4 included *S. sonnei* isolates from Thailand and Nepal with variable antimicrobial susceptibility patterns [see Additional file 1].

### Replicon typing

*S. sonnei* isolates were classified for plasmid incompatibility by PCR-based replicon typing (PBRT) with 21 primer pairs (B/O, FIC, A/C, P, T, K/B, W, FIIA, FIA, FIIB, Y, I1, Frep, X, HI1, N, HI2, L/M, ColE, IncR and IncU plasmid. All 38 isolates from Bhutan, Nepal, and Thailand carried the ColE plasmid. Nine isolates from Bhutan and one isolate from Thailand carried B/O plasmid. One isolate from Bhutan and three isolates from Thailand carried the I1 plasmid (Table 2).

### PCR and sequencing analysis

The *gyrA* genes of the 38 *S. sonnei* isolates from Bhutan, Nepal and Thailand were amplified. The sequence analysis of the 470 bp amplified products from 29 *S. sonnei* isolates from Bhutan and one isolate from Nepal revealed the presence of two point mutations in the GyrA subunit (Ser83Leu and Asp87Gly) (Table 2). However, no amplified products were observed for the *mph*A gene in all 38 *S. sonnei* isolates compared to an azithromycin resistant Enteroaggregative *E. coli* control.

All 38 *S. sonnei* isolates had amplified products of class 1 integrons of approximately 700 bp. By DNA sequencing, the amplified products of 5 selected *S. sonnei* isolates from Bhutan showed no homologies to published gene cassettes of class 1 integrons but had 99% identity of conserved hypothetical protein *S. sonnei* Ss046 in the NCBI database. Thus, *S. sonnei* in Bhutan did not contain resistance genes in class 1 integrons. The class 2 integrons of 38 *S. sonnei* isolates were also amplified. The amplified products of 29 isolates from Bhutan and 3 isolates from Nepal were of similar size (2.4 Kb) but differed from the class 2 integrons in the 3 Thailand isolates (~3-4 Kb). The DNA sequence alignment of class 2 integrons from 5 selected *S. sonnei* isolates from Bhutan had homology to dihydrofolate reductase (*dfrA1*)*,* streptothricin acetyltransferase (*sat1*) and aminiglycoside adenyltransferase (*aadA1*) associated with resistance to trimethoprim, streptothricin and streptomycin/spectinomycin, respectively. Moreover, RFLP of amplified products of the class 2 integrons in all 38 isolates with *Xba*I, yielded the same DNA restricted-fragments of approximately 1,600 bp and 800 bp in 29 *S. sonnei* isolates from Bhutan and 1 isolate from Nepal. The other Nepal and Thailand isolates had uncut DNA or undetectable bands.

## Discussion

Over the last few years, MDR patterns in *Shigella* spp*.* have been reported in many countries [20]. In this study, all *S. sonnei* isolates from children in Thimphu, Bhutan in June and July, 2011 were resistant to multiple drugs like ciprofloxacin, nalidixic acid, trimethoprim-sulfamethoxazole, streptomycin and tetracycline. The MDR patterns of the *S. sonnei* from Bhutan differed from neighboring countries. In India, antimicrobial resistance in *S. sonnei* is more common than in other enteric bacteria with resistant to azithromycin, ciprofloxacin, furazolidone, nalidixic acid, norfloxacin, ofloxacin, trimethoprim-sulfamethoxazole, tetracycline [21]. In Bangladesh, most of *S. sonnei* isolates were resistant to nalidixic acid, sulfamethoxazole-trimethoprim, ampicillin, streptomycin and tetracycline [22]. In China, the antimicrobial resistance profiles of *S. sonnei* were nalidixic acid, piperacilline and ciprofloxacin [23]. In Nepal, *S. sonnei* isolates were resistant to azithromycin, ciprofloxacin, ceftriaxone, ampicillin, nalidixic acid and trimethoprim-sulfamethoxazole [24].

*Shigellae* usually harbor various plasmids, such as those required for bacterial invasion into the host intestinal epithelial cells and antibiotic resistance, which may range in number from two to as many as ten in one strain [25]. The PFGE and plasmid patterns of *S. sonnei* from Thimphu, Bhutan suggested clonality. *S. sonnei* in Thimphu, Bhutan carried the typical number of variable plasmid sizes from 0.35 Kb to 98 Kb. These *S. sonnei* also contained the typical number of plasmids from seven to ten in one strain. The ability to recognize and categorize plasmids in homogeneous groups on the basis of their phylogenetic relatedness is helpful to analyze their distribution in nature and their relationship to host cells and to discover their evolutionary origins [26]. Classification of plasmids into incompatibility groups is desirable because specific plasmid types have been associated with virulence and/or antimicrobial resistance [15]. The plasmid replicon typing results showed that all *S. sonnei* isolates from Thimphu, Bhutan carried ColE plasmid as the same as control isolates from Nepal and Thailand. ColE plasmid which contains genes that code for colicin protein (bacteriocin protein) that can kill other bacteria and can replicate in the absence of de novo protein synthesis [26,27]. Furthermore, ColE plasmids co-resided with B/O or I1 in several *S. sonnei* isolates. The simultaneous presence of the ColE plasmid with additional plasmids belonging to the I1, B/O or A/C groups within the same parental strain suggested that the latter plasmids can participate in the mobilization of the ColE plasmids. B/O and I1 plasmid are plasmid incompatibility (Inc) groups capable of carrying transfer, MDR, and virulence functions [28].

Previous studies reported on the frequent incidence of class 2 integrons in *Shigella* isolates worldwide. DNA sequencing of the class 2 integrons in this study showed homologies with class 2 integrons but no homologies to published gene cassettes of class 1 integrons. In this study, *S. sonnei* from Thimphu, Bhutan carried three classic gene cassettes (2,433 bp) in transposon Tn7 corresponding to previous results. *S. sonnei* isolates from Korea carried class 2 integrons (2,224 bp) that consist of Tn7 with *dfrA1*, *sat1* and *aadA1*[29]. In Japan, two types of class 2 integrons in *S. sonnei* isolates were previously identified. One was the classical type (2,158 bp) with *dfrA1*, *sat1* and *aadA1*. The other type was shorter (1,313 bp) and carried only two gene cassettes with *dfrA1* and *sat1*[20]. In China, a 3,361 bp DNA fragment was found in *S. sonnei* with gene cassette arrays in this class 2 integrons of *dfrA1*, *sat1*, *aadA1* and *orfX*[17].

Nalidixic acid resistance among enteric bacteria is related to the presence of a single amino-acid substitution at either position 83 or position 87 of GyrA, while resistance to ciprofloxacin is related to the presence of at least one additional substitution in GyrA [30]. In this study, *S. sonnei* in Thimphu, Bhutan presented two mutations at position 83 (Serine to Leucine) and position 87 (Aspartic acid to Glycine) related to NA and CIP resistance. These two mutations conferred extremely high resistance to NA in *S. sonnei* in Bhutan that has been reported previously from *S. sonnei* isolates in Japan [31]. The expression of *mphA* gene is related to the decrease of AZM susceptibility [19]. In the USA, isolates with a susceptibility at 8 mg/L lacked *mphA* gene but isolates with higher MICs (>64 mg/L) to AZM reportedly contained a plasmid-encoded *mphA* gene [19]. In this study, MIC range of AZM was 3.0-4.0 μg/ml and PCR results showed that *mph*A gene was not present.

## Conclusions

This study described the recent antimicrobial resistance among *S. sonnei* strains isolated from Thimphu, Bhutan in June and July, 2011 and molecular characterization of these *S. sonnei*. Plasmid patterns, PFGE and uniform MDR suggest that these 29 *S. sonnei* isolates are closely related. All *S. sonnei* isolates carried common ColE plasmid that co-resided with B/O or I1 plasmid in many isolates. Further study and comparison with other isolates from the region may provide information on the development and continued spread of antibiotic resistance in *S. sonnei*.

## Competing interests

The authors declare that they have no competing interests.

## Authors’ contributions

SR performed laboratory assay on molecular typing, data analysis and prepared manuscript. TD coordinated laboratory work between Bhutan and AFRIMS, and performed laboratory work in Bhutan. PP and PN performed laboratory assay on molecular typing and data analysis. OS provided comments and suggestions on molecular methods and reviewed the manuscript. KP performed laboratory on antimicrobial susceptibility testing and data analysis. KPT provided stool samples from Jigme Dorji Wangchuk National Referral Hospital to the Public Health Laboratory in Bhutan. SW and LB contributed to study design and protocol development, and coordinated laboratory infrastructure development and quality control programs in the Public Health Laboratory. CJM oversaw any laboratory work done in the Department of Enteric Diseases at AFRIMS and reviewed the manuscript. All authors’ read and approved the final manuscript.

## Supplementary Material

Additional file 1 **The dendrogram of plasmid profile.** The dendrogram obtained by cluster analysis of the plasmid patterns of *S. sonnei* from Bhutan, Nepal and Thailand combined with antibiotic susceptibility. Click here for file
