# Supplementary material for: Molecular characterization and PCR-based replicon typing of multidrug resistant Shigella sonnei isolates from an outbreak in Thimphu, Bhutan
Source: BMC Res Notes. 2014 Feb 20;7:95. doi: 10.1186/1756-0500-7-95 (PMC3936901; doi:10.1186/1756-0500-7-95)
Supplement: Additional file 1 — The dendrogram of plasmid profile. The dendrogram obtained by cluster analysis of the plasmid patterns of S. sonnei from Bhutan, Nepal and Thailand combined with antibiotic susceptibility. [file 1756-0500-7-95-S1.pdf]

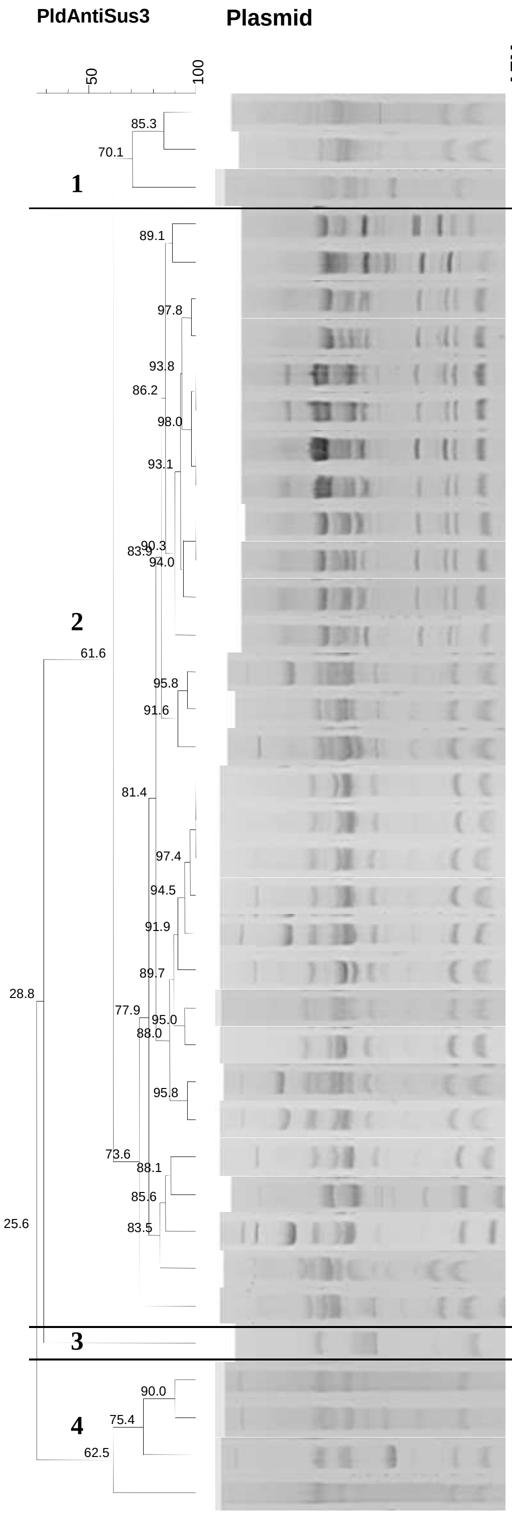

AntiSus3

AZM	NA	CIP	AM	SXT	CRO	S	TE			
S	R	S	S	R	S	R	R	BH-07-0111	Bharatpur	Nepal
S	R	S	S	R	S	R	R	KH-08-0362	Kanti	Nepal
S	R	S	S	R	ND	R	R	PN-08-375	Pisanulok	Thailand
S	R	R	S	R	S	R	R	JH-11-102	Thimphu	Bhutan
S	R	R	S	R	S	R	R	JH-11-134	Thimphu	Bhutan
S	R	R	S	R	S	R	R	JH-11-106	Thimphu	Bhutan
S	R	R	S	R	S	R	R	JH-11-110	Thimphu	Bhutan
S	R	R	S	R	S	R	R	JH-11-108	Thimphu	Bhutan
S	R	R	S	R	S	R	R	JH-11-132	Thimphu	Bhutan
S	R	R	S	R	S	R	R	JH-11-105	Thimphu	Bhutan
S	R	R	S	R	S	R	R	JH-11-131	Thimphu	Bhutan
S	R	R	S	R	S	R	R	JH-11-047	Thimphu	Bhutan
S	R	R	S	R	S	R	R	JH-11-095	Thimphu	Bhutan
S	R	R	S	R	S	R	R	JH-11-109	Thimphu	Bhutan
S	R	R	S	R	S	R	R	JH-11-137	Thimphu	Bhutan
S	R	R	S	R	S	R	R	JH-11-189	Thimphu	Bhutan
S	R	R	S	R	S	R	R	JH-11-209	Thimphu	Bhutan
S	R	R	S	R	S	R	R	JH-11-198	Thimphu	Bhutan
S	R	R	S	R	S	R	R	JH-11-135	Thimphu	Bhutan
S	R	R	S	R	S	R	R	JH-11-180	Thimphu	Bhutan
S	R	R	S	R	S	R	R	JH-11-181	Thimphu	Bhutan
S	R	R	S	R	S	R	R	JH-11-162	Thimphu	Bhutan
S	R	R	S	R	S	R	R	JH-11-147	Thimphu	Bhutan
S	R	R	S	R	S	R	R	JH-11-161	Thimphu	Bhutan
S	R	R	S	R	S	R	R	BH-08-1075	Bharatpur	Nepal
S	R	R	S	R	S	R	R	JHC-11-110	Thimphu	Bhutan
S	R	R	S	R	S	R	R	JH-11-182	Thimphu	Bhutan
S	R	R	S	R	S	R	R	JHC-11-108	Thimphu	Bhutan
S	R	R	S	R	S	R	R	JH-11-175	Thimphu	Bhutan
S	R	R	S	R	S	R	R	JHC-11-091	Thimphu	Bhutan
S	R	R	S	R	S	R	R	JHC-11-135	Thimphu	Bhutan
S	R	R	S	R	S	R	R	JH-11-224	Thimphu	Bhutan
S	R	R	S	R	S	R	R	JH-11-223	Thimphu	Bhutan
S	S	S	S	S	S	R	S	SH-07-0096	Sukraraj	Nepal
S	R	S	R	R	ND	R	R	PN-08-298	Pisanulok	Thailand
S	R	S	R	R	ND	R	R	PN-08-349	Pisanulok	Thailand
S	R	S	R	R	ND	R	R	CR-08-236	Chiangrai	Thailand
S	S	S	R	R	ND	R	R	CR-08-301	Chiangrai	Thailand

Additional file 1 Dendogram obtained by cluster analysis of the plasmid patterns of *S. sonnei* from Bhutan, Nepal and Thailand combined with antibiotic susceptibility. AZM, azithromycin; NA, nalidixic acid; CIP, ciprofloxacin; AM, ampicillin; SXT, trimethoprim-sulfamethoxazole; CRO, ceftriaxone; S, Streptomycin; TE, tetracycline; S, susceptible; R, resistant; ND, not determined.
